# The Outcomes of Minimally Invasive versus Open Posterior Approach Spinal Fusion in Treatment of Lumbar Spondylolisthesis: The Current Evidence from Prospective Comparative Studies

**DOI:** 10.1155/2017/8423638

**Published:** 2017-01-05

**Authors:** Ai-Min Wu, Chun-Hui Chen, Zhi-Hao Shen, Zhen-Hua Feng, Wan-Qing Weng, Shu-Min Li, Yong-Long Chi, Li-Hui Yin, Wen-Fei Ni

**Affiliations:** ^1^Department of Orthopedics, Second Affiliated Hospital of Wenzhou Medical University, Second Medical College of Wenzhou Medical University, Zhejiang Spine Center, Wenzhou, Zhejiang, China; ^2^Department of Orthopedics, Hainan Medical College, Haikou, Hainan, China; ^3^Laboratory of Internal Medicine, The First Affiliated Hospital of Wenzhou Medical University, Wenzhou, Zhejiang, China

## Abstract

*Purpose*. To investigate the evidence of minimally invasive (MI) versus open (OP) posterior lumbar fusion in treatment of lumbar spondylolisthesis from current prospective literatures.* Methods*. The electronic literature database of Pubmed, Embase, and Cochrane library was searched at April 2016. The data of operative time, estimated blood loss and length of hospital stay, visual analog scale (VAS) of both lower back pain and leg pain, Oswestry disability index (ODI), SF-36 PCS (physical component scores) and SF-36 MCS (mental component scores), complications, fusion rate, and secondary surgery were extracted and analyzed by STATA 12.0 software.* Results*. Five nonrandom prospective comparative studies were included in this meta-analysis. The meta-analysis showed that the MI group had a significantly longer operative time than OP group, less blood loss, and shorter hospital stay. No significant difference was found in back pain, leg pain, ODI, SF-36 PCS, SF-36 MCS, complications, fusion rate, and secondary surgery between MI and OP groups.* Conclusion*. The prospective evidence suggested that MI posterior fusion for spondylolisthesis had less EBL and hospital stay than OP fusion; however it took more operative time. Both MI and OP fusion had similar results in pain and functional outcomes, complication, fusion rate, and secondary surgery.

## 1. Introduction

With the help of radiographic and endoscopic system and special surgical tools, the minimally invasive posterior lumbar surgery was developed and worldwide popularly in last decades [1, 2]. It was reported the minimally invasive spinal surgery techniques had advantages of shorter skin wound incision, less muscle trauma, less blood loss, and hospital stay [3–5].

Currently, the minimally invasive (MI) lumbar spinal fusion techniques including MI posterior lumbar interbody fusion [6], MI transforaminal lumbar interbody fusion [7, 8], MI posterolateral lumbar fusion, MI lateral lumbar fusion [9], MI oblique lumbar interbody fusion, and MI anterior lumbar interbody fusion. The posterior approach permits the decompression and discectomy directly and does not have complications of vessel, hypogastric sympathetic plexus, and ureter injury, which may be caused by anterior approach [10–12], and is most widely used nowadays [13].

And the previous systematic review and meta-analysis in literatures showed that MI transforaminal lumbar interbody fusion appears similar safety and efficacy to open transforaminal lumbar interbody fusion and associated with lower blood loss and infection rates for general degenerative lumbar disease patient [14, 15].

However, spondylolisthesis is one of the most lumbar spinal disorders and may be caused by isthmic or degeneration. The symptoms of spondylolisthesis include low back pain and leg pain, decreasing walk ability, and neurogenic claudication. Surgical interventions were recommended when the symptoms could not be relieved by conservative treatment [16–18]. The difference of spondylolisthesis to other degenerative lumbar diseases (such as lumbar stenosis without spondylolisthesis and lumbar disc herniated) is that in spondylolisthesis patients the vertebrae will be slipped anteriorly. The traditional open spinal fusion, which performed laminectomy to completely decompression the spinal canal and nerve root, was recognized as one of the “gold standard” methods in treatment of spondylolisthesis and had credible pain relief and function improvement [19, 20]. MI technique may be hard to achieve completely decompression because of the limited vision; therefore, the clinical efficacy and safety of minimally invasive posterior spinal fusion in treatment of lumbar spondylolisthesis are still controversial. In this study, we aim to provide the best evidence from current prospective comparative studies for surgeons and researchers.

## 2. Methods

This systematic review and meta-analysis was done according to the preferred reporting items for systematic review and meta-analyses (PRISMA) guidelines (Checklist S1 in Supplementary Material available online at https://doi.org/10.1155/2017/8423638) [21]. No primary personal data will be collected; therefore no additional ethical approval needs to be obtained.

### 2.1. Search Strategy

Two authors (Chun-Hui Chen and Zhi-Hao Shen) independently searched the electronic literature database of Pubmed, Embase, and Cochrane library, without language limitation at April 2016. The key words were used as follows: posterior lumbar interbody fusion, transforaminal lumbar interbody fusion, posterolateral lumbar fusion, posterior lumbar fusion, posterior lumbar arthrodesis, minimally invasive lumbar fusion, minimally invasive fusion, spondylolisthesis, isthmic spondylolisthesis, and degenerative spondylolisthesis. One of search strategy developed with comprehensive use of keywords performed in Pubmed was showed in Table S1. Related articles and reference lists were searched to avoid original miss.

### 2.2. Eligibility Criteria

The study was included in this meta-analysis if it was (1) prospective randomized controlled trial (RCT) or nonrandomized prospective comparative study; (2) it compared the clinical outcomes of minimally invasive posterior approach lumbar fusion versus traditional open posterior approach lumbar fusion; (3) the participants were spondylolisthesis (including isthmic and degenerative spondylolisthesis); (4) it was with a follow-up term of at least 12 months.

Exclusion criteria were as follows: (1) respective studies, case series, case report, and review articles; (2) follow-up of less than 12 months; (3) duplicated publications from the same hospital or research center.

### 2.3. Selection of Literature

**Figure 1:**
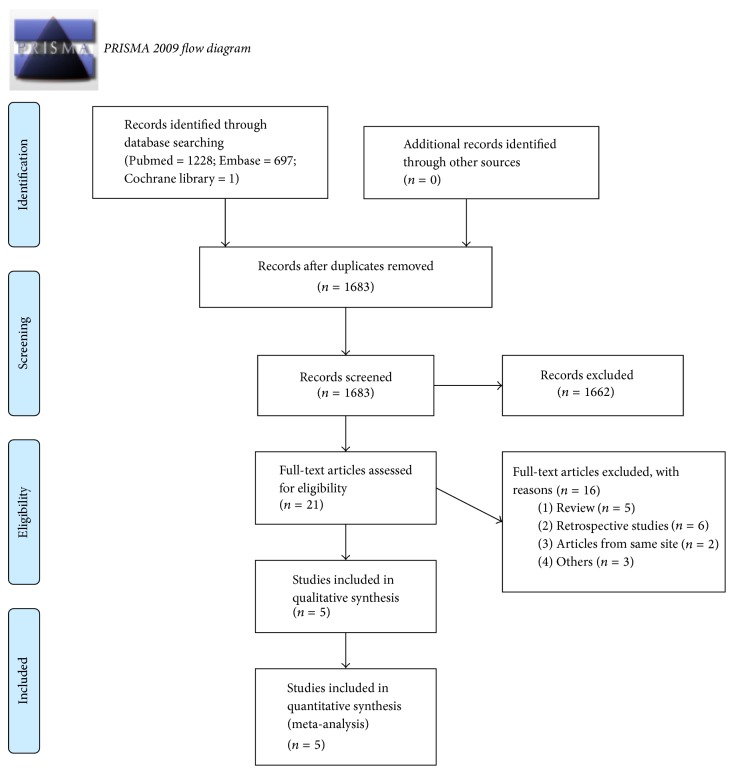
Flowchart of the study selection process. From [21]. For more information, visit http://www.prisma-statement.org/.

We used the PRISMA flow diagram to select the included studies (Figure 1); the results of literature search were imported into the software Endnote X4. Two authors (Zhen-Hua Feng and Wan-Qing Weng) independently assessed the potentially eligible studies. Firstly, the titles and abstracts were screened to exclude the duplicated and apparently irrelevant ones or those that do not meet our inclusion criteria. After then, the remaining potential studies were full-text downloaded and reviewed. Any disagreement between two above authors was sent and discussed with the third independent author (Ai-Min Wu).

### 2.4. Data Extraction

Two reviewers (Chun-Hui Chen and Shu-Min Li) independently extracted data, and the third reviewer (Wen-Fei Ni) checked the consistency between them. A standard form was used; the extracted items included the following: (1) the general study information, for example, the authors, publishing date, country, name of investigate site, study design, sample size, age, gender, index levels, follow-up term; (2) perioperative parameters, including operative time, estimated blood loss, X-ray exposure, and length of hospital stay; (3) clinical outcomes, including visual analog scale (VAS) of both lower back pain and leg pain, Oswestry disability index (ODI), SF-36 PCS (physical component scores), and SF-36 MCS (mental component scores); (4) complications, nonfusion rate, and secondary surgery; the complications included dural tear, wound infection, screw or rod fracture, graft dislodgement, epidural hematoma, and adjacent disc disease. For continuous outcomes, we extracted the mean and SD (standard deviation) and participant number will be extracted. For dichotomous outcomes, we extracted the total numbers and the numbers of events of both groups. The data in other forms was recalculated when possible to enable pooled analysis.

### 2.5. Quality Assessment of Included Studies

The methodological index for nonrandomized studies (MINORS) was used to assess the quality of the included studies [22, 23]. Twelve items were scored as “0” (not reported), “1” (reported but inadequate), or “2” (reported and adequate). Two reviewers (Ai-Min Wu and Yong-Long Chi) independently assessed the quality of the included studies.

### 2.6. Statistical Analysis

The data was collected and input into the STATA software (version 12.0; StataCorp, College Station, TX) for meta-analysis. Random-effects model was used to combine the data from individual studies. Relative risk (RR) was calculated for dichotomous outcomes such as complications, nonfusion, and secondary surgery. Standard mean difference (SMD) was calculated for continuous outcomes such as operative time, estimated blood loss, length of hospital stay, and clinical parameters. Heterogeneity was assessed using the *x*^2^ and *I*^2^. We defined the acceptable heterogeneity by *p* value of *x*^2^ test > 0.10 and *I*^2^ < 50%. For heterogeneity data, sensitivity analysis was involved to remove one study and evaluate whether the other results would be markedly affected.

## 3. Results

### 3.1. Included Studies

A total 1926 potential records were identified through Medline (*n* = 1228), Embase (*n* = 697), and Cochrane library (*n* = 1). The list of articles were input into software endnote X4, and then 243 duplicate articles were excluded, after titles and abstracts screened, leaving 21 full-text articles to be assessed for eligibility, and 16 were excluded for reasons of “the papers were review or retrospective studies or from same investigation site” and some other reasons (details were showed in Figure 1). Finally, five nonrandom prospective comparative studies [24–28] were included in this meta-analysis. The procedure of literatures selection was showed in Figure 1 (PRISMA flow diagram).

### 3.2. Characteristics and Qualifications of Included Studies

The characteristics of all five included studies were summarized and shown in Table 1. All the five included studies [24–28] were prospective comparative studies without random. They were from five different countries (Australia, China, Germany, Japan, and USA) and all of them were published after 2010. Total of 184 participants in MI group and 182 in OP group were included in this meta-analysis. The methodological quality assessment of the five included studies was summarized in Table 2. The scores ranged from 18 to 20 with a median value of 19. The summary of outcomes of included studies was shown in Table 3.

### 3.3. Perioperative Parameters

**Figure 2:**
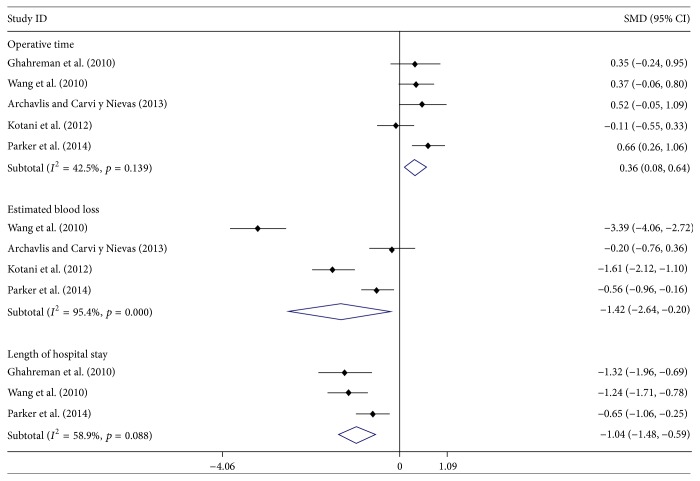
Forest plot showing the meta-analysis of operative time, estimated blood loss, and length of hospital stay.

All five studies [24–28] reported the operative time data of both groups; the meta-analysis showed that the MI group had a significantly longer operative time than OP group, with SMD = 0.36 (95% CI: 0.08, 0.64). Four studies [25–28] reported the data of estimated blood loss; the meta-analysis showed that the MI group had a significantly less blood loss than the OP group, with SMD = −1.42 (95% CI: −2.64, −0.20). Three studies [24, 25, 27] reported the length of hospital stay; the meta-analysis showed that the MI group had a significantly shorter hospital stay than the OP group, with SMD = −1.04 (95% CI: −1.48, −0.59) (Figure 2). Heterogeneity was observed in data of estimated blood loss (*I*^2^ = 95.4%, *p* = 0.000) and length of hospital stay (*I*^2^ = 58.9%, *p* = 0.088); sensitivity analysis found that there was no significant change when any study was omitted (Figures S1 and S2).

### 3.4. Clinical Outcomes

**Figure 3:**
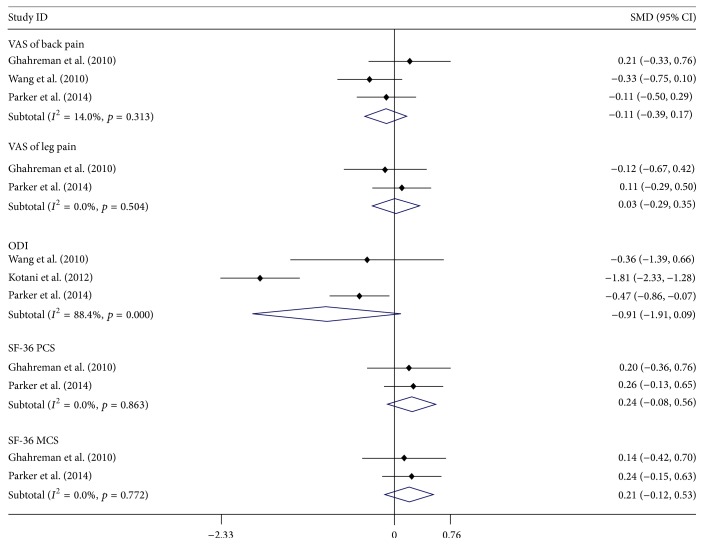
Forest plot showing the meta-analysis of visual analogue scale (VAS) scores for low back pain and leg pain, the Oswestry disability index, SF-36 PCS (physical component scores), and SF-36 MCS (mental component scores).

Three studies [24, 25, 28] reported the data of back pain and two studies [24, 28] reported leg pain; the meta-analysis showed there was no significant difference between both MI and OP groups, SMD of back pain = −0.11 (95% CI: −0.39, 0.17) and SMD of leg pain = 0.03 (95% CI: −0.29, 0.35). Three studies [25, 27, 28] reported the data of ODI, and the meta-analysis showed that there was no significant difference between both MI and OP groups, with SMD of ODI = −0.91 (95% CI: −1.91, 0.09). Two studies [24, 28] reported SF-36 PCS and MCS, the meta-analysis showed that there was no significant difference between both MI and OP groups, SMD of SF-36 PCS = 0.24 (95% CI: −0.08, 0.56) and SMD of SF-36 MCS = 0.21 (95% CI: −0.12, 0.53) (Figure 3). Heterogeneity was observed in data of ODI, with *I*^2^ = 88.4%, *p* = 0.000; sensitivity analysis found that there was no significant change when any study was omitted (Figure S3).

### 3.5. Adverse Events

**Figure 4:**
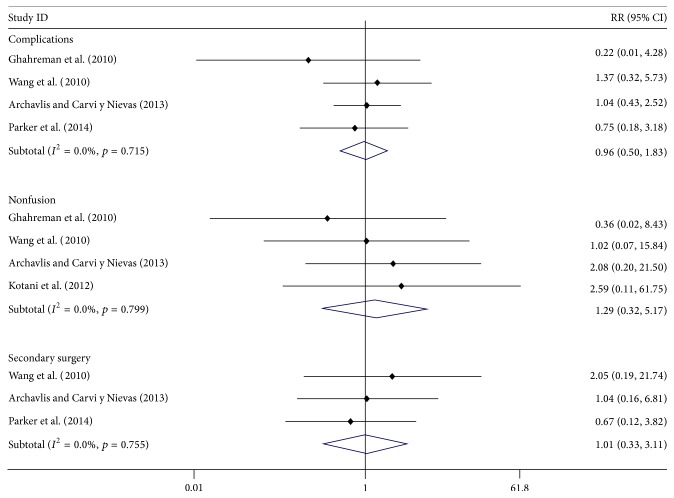
Forest plot showing the meta-analysis of complications, rate of fusion, and secondary surgery.

Four studies [24–26, 28] reported the data of complications; the meta-analysis showed that there was no significant difference between both MI and OP groups, with RR = 0.96 (95% CI: 0.50, 1.83). Four studies [24–27] reported the data of nonfusion rate; the meta-analysis showed that there was no significant difference between both MI and OP groups, with RR = 1.29 (95% CI: 0.32, 5.17). And three studies [25, 26, 28] reported the data of secondary surgery; the meta-analysis showed that there was no significant difference between both MI and OP groups, with RR = 1.01 (95% CI: 0.33, 3.11) (Figure 4). No obvious heterogeneity was observed in data of complications, nonfusion, and secondary surgery.

## 4. Discussion

The technique of posterior/posterolateral lumbar fusion had more than 100 years' history [29], there are many different kinds of lumbar fusion now, and they are widely used in treatment of lumbar disc herniation, lumbar instability, and spondylolisthesis [13, 30–32]. To reduce the operative trauma [33, 34], Foley et al. reported using the miniopen tubule microsurgical approach with percutaneous pedicle screw fixation to achieve lumbar arthrodesis [1, 35]. The minimally invasive technique was modified and widespread in last decades.

Khan et al. [15] performed a meta-analysis of MI-TLIF versus open TLIF and found that the MI-TLIF can significantly reduce the blood loss, length of hospital stay, and complications; however, the fusion rate and operative time was similar. Another meta-analysis [14] found that the MI-TLIF not only reduced the blood loss more than open TLIF but also had significantly lower VAS of back pain and ODI scores.

In our this meta-analysis, only the spondylolisthesis patients were included, and we found that the MI technique can significantly reduce the estimated blood loss and length of hospital stay; however, it took more operative time. The decompression process of lumbar spondylolisthesis may need more time because of the limited space and vision, and the minimally invasive technique also needs a longer learning curve for surgeons [36, 37]. Another inconsistency to Phan et al.' meta-analysis that we did not find significant difference in VAS of back pain and ODI scores between MI and OP groups. The patients with spondylolisthesis had the similar results in back and leg pain, ODI, and SF-36 scores, as well as complications, fusion rate, and secondary surgery between MI and OP groups.

Currently, MI lumbar fusion is mainly used in treatment of lower grade spondylolisthesis [25, 38]. Whether the MI lumbar fusion can be used in treatment of high-grade spondylolisthesis is still a controversial subject. Quraishi and Rampersaud reported that they use the minimally invasive bilateral transforaminal lumbar interbody fusion to treat high-grade isthmic spondylolisthesis [39], with estimated blood loss less than 100 ml, and about 150 minutes' operating time. The slip percentage improved from 68% preoperatively to 28% postoperatively. Because the estimated blood loss is more than 1000 ml when high-grade spondylolisthesis patients underwent traditional open TLIF [40], they suggested the MI technique may have more advantage in blood loss in high-grade spondylolisthesis. The evidence that MI fusion is better for high-grade spondylolisthesis still needs further research.

*Strength and Limitation of This Study*. This study had many strengths; all of the included studies in this meta-analysis were prospective studies, which therefore overcomes the shortcomings of recall or selection bias in retrospective studies [41]. The methodological index for nonrandomized studies (MINORS) was used to assess the quality of the included studies, which had minimized scores of 18, the score range of 18 to 20.

However, there were still some limitations of present study, none of them was randomized control trials, the sample size was not very large, and the duration of follow-up was less than 5 years. Therefore, we suggested further long-term, larger sample size, and randomized control trials to be conducted.

## 5. Conclusions

In summary, our present meta-analysis base prospective comparative studies suggested that MI posterior fusion for spondylolisthesis had less EBL and hospital stay than OP fusion; however it took more operative time. Both MI and OP fusion had similar results in pain and functional outcomes, complication, fusion rate, and secondary surgery.

## Supplementary Material

Checklist S1: PRISMA 2009 checklist. Table S1: The developed search strategy performed in database of Pubmed. Its already right here. Figure S1–3: The results of sensitivity analysis.

## Figures and Tables

**Table 1:** The characteristics of the included studies.

Authors	Ghahreman et al.	Wang et al.	Archavlis and Carvi y Nievas	Kotani et al.	Parker et al.
Year	2010	2010	2013	2012	2014
Study design	PCT	PCT	PCT	PCT	PCT
Country	Australia	China	Germany	Japan	USA
Age (years)	MI: 53 (40–61) OP: 60 (48–63)	MI: 47.9 ± 8.5 OP: 53.2 ± 10.6	MI: 67 ± 8 OP: 68 ± 7	MI: 63 ± 9 OP: 66 ± 9	MI: 53.5 ± 12.5 OP: 52.6 ± 11.6
Number of participants	MI: 25 OP: 27	MI: 42 OP: 43	MI: 24 OP: 25	MI: 43 OP: 37	MI: 50 OP: 50
Gender					
Male	MI: 12; OP: 13	MI: 13; OP: 16	MI: 14; OP: 10	MI: 14; OP: 12	MI: 16; OP: 18
Female	MI: 13; OP: 14	MI: 29; OP: 27	MI: 17; OP: 8	MI: 29; OP: 25	MI: 34; OP: 32
Index levels					
L3-4	MI: 0; OP: 2	MI: 3; OP: 3	MI: 2; OP: 1	4	MI: 4; OP: 3
L4-5	MI: 11; OP: 10	MI: 21; OP: 23	MI: 16; OP: 17	76	MI: 32; OP: 30
L5-S1	MI: 11; OP: 15	MI: 18; OP: 17	MI: 6; OP: 7	—	MI: 14; OP: 17
L4-S1	MI: 3; OP: 0	—	—	—	—
Follow-up term (months)	12	26 (13–35)	24	MI: 32 (24–49); OP: 40 (24–60)	24

MI: minimally invasive TLIF group; OP: open TLIF group; PCT: prospective comparative trials.

**Table 2:** Quality assessment of five included studies.

Methodological item for nonrandomised studies	Ghahreman et al.	Wang et al.	Archavlis and Carvi y Nievas	Kotani et al.	Parker et al.
(1) A clearly stated aim	2	2	2	2	2
(2) Inclusion of consecutive patients	1	2	2	1	1
(3) Prospective collection of data	2	2	2	2	2
(4) Endpoints appropriate to the aim of the study	2	2	2	2	2
(5) Unbiased assessment of the study end point	0	0	0	0	0
(6) Follow-up period appropriate to the aim of the study	1	1	2	2	2
(7) Loss to follow-up less than 5%	2	2	2	2	2
(8) Prospective calculation of the study size	0	0	0	0	0
(9) An adequate control group	2	2	2	2	2
(10) Contemporary groups	2	2	2	2	2
(11) Baseline equivalence of groups	2	2	2	2	2
(12) Adequate statistical analyses	2	2	2	2	2
Total scores	18	19	20	19	19

**Table 3:** The summary of outcomes of included studies.

Authors	Ghahreman et al.	Wang et al.	Archavlis and Carvi y Nievas	Kotani et al.	Parker et al.
Operative time (mins)	MI: 236 ± 68 OP: 213 ± 67	MI: 156 ± 32 OP: 145 ± 27	MI: 220 ± 48 OP: 190 ± 65	MI: 172 ± 33 OP: 176 ± 37	MI: 284 ± 95 OP: 230 ± 67
EBL	—	MI: 264 ± 89 OP: 673 ± 145	MI: 185 ± 140 OP: 255 ± 468	MI: 184 ± 36 OP: 453 ± 243	MI: 233 ± 229 OP: 383 ± 305
Hospital stay	MI: 4 ± 1.58 OP: 6.67 ± 2.36	MI: 10.6 ± 2.5 OP: 14.6 ± 3.8	—	—	MI: 3 ± 1.53 OP: 4 ± 1.53
Back pain VAS	MI: 2.67 ± 3.14 OP: 2 ± 3.13	MI: 0.92 ± 0.5 OP: 1.1 ± 0.6	—	—	MI: 3.3 ± 2.9 OP: 3.6 ± 2.8
Leg pain VAS	MI: 1.33 ± 2.36 OP: 1.67 ± 3.13	—	—	—	MI: 3 ± 3 OP: 2.7 ± 2.6
ODI	—	MI: 10.8 ± 3.3 OP: 12.2 ± 3.9	—	MI: 12.8 ± 13.3 OP: 36.5 ± 12.9	MI: 11 ± 9.4 OP: 15.6 ± 10.3
SF-36 PCS	MI: 64.33 ± 40.98 OP: 56.67 ± 35.37	—	—	—	MI: 44.3 ± 11.2 OP: 41.3 ± 11.8
SF-36 MCS	MI: 76.67 ± 18.87 OP: 72.67 ± 36.16	—	—	—	MI: 54.5 ± 10.8 OP: 52 ± 10.1
Complications	MI: 0/25 OP: 2/27	MI: 4/42 OP: 3/43	MI: 7/24 OP: 7/25	—	MI: 3/50 OP: 4/50
Nonfusion	MI: 0/25 OP: 1/27	MI: 1/42 OP: 1/43	MI: 2/24 OP: 1/25	MI: 1/43 OP: 0/37	—
Secondary Surgery	—	MI: 2/42 OP: 1/43	MI: 2/24 OP: 2/25	—	MI: 2/50 OP: 3/50

MI: minimally invasive surgery group; OP: open surgery group; EBL: estimated blood loss; VAS: visual analog scale; ODI: Oswestry disability index; SF-36 PCS: Short Form-36 physical component scores; SF-36 MCS: Short Form-36 mental component scores.

## References

[1] Foley K. T., Holly L. T., Schwender J. D. (2003). Minimally invasive lumbar fusion. *Spine*.

[2] Kim T. T., Johnson J. P., Pashman R., Drazin D. (2016). Minimally invasive spinal surgery with intraoperative image-guided navigation. *BioMed Research International*.

[3] Fan S., Hu Z., Zhao F., Zhao X., Huang Y., Fang X. (2010). Multifidus muscle changes and clinical effects of one-level posterior lumbar interbody fusion: minimally invasive procedure versus conventional open approach. *European Spine Journal*.

[4] Tian N.-F., Wu Y.-S., Zhang X.-L., Xu H.-Z., Chi Y.-L., Mao F.-M. (2013). Minimally invasive versus open transforaminal lumbar interbody fusion: a meta-analysis based on the current evidence. *European Spine Journal*.

[5] Nie H., Zeng J., Song Y. (2016). Percutaneous endoscopic lumbar discectomy for L5-S1 disc herniation via an interlaminar approach versus a transforaminal approach: a prospective randomized controlled study with 2-year follow up. *Spine*.

[6] Sidhu G. S., Henkelman E., Vaccaro A. R. (2014). Minimally invasive versus open posterior lumbar interbody fusion: a systematic review. *Clinical Orthopaedics and Related Research*.

[7] Karikari I. O., Isaacs R. E. (2010). Minimally invasive transforaminal lumbar interbody fusion: a review of techniques and outcomes. *Spine*.

[8] Choi W., Kim J., Ryu K., Hur J., Seong J. (2016). Minimally invasive transforaminal lumbar interbody fusion at L5-S1 through a unilateral approach: Technical feasibility and outcomes. *BioMed Research International*.

[9] Ozgur B. M., Aryan H. E., Pimenta L., Taylor W. R. (2006). Extreme lateral interbody fusion (XLIF): a novel surgical technique for anterior lumbar interbody fusion. *Spine Journal*.

[10] Goz V., Weinreb J. H., Schwab F., Lafage V., Errico T. J. (2014). Comparison of complications, costs, and length of stay of three different lumbar interbody fusion techniques: an analysis of the Nationwide Inpatient Sample database. *Spine Journal*.

[11] Jiang S.-D., Chen J.-W., Jiang L.-S. (2012). Which procedure is better for lumbar interbody fusion: anterior lumbar interbody fusion or transforaminal lumbar interbody fusion?. *Archives of Orthopaedic and Trauma Surgery*.

[12] Baker J. K., Reardon P. R., Reardon M. J., Heggeness M. H. (1993). Vascular injury in anterior lumbar surgery. *Spine*.

[13] Yoshihara H., Yoneoka D. (2015). National trends in the surgical treatment for lumbar degenerative disc disease: United States, 2000 to 2009. *Spine Journal*.

[14] Phan K., Rao P. J., Kam A. C., Mobbs R. J. (2015). Minimally invasive versus open transforaminal lumbar interbody fusion for treatment of degenerative lumbar disease: systematic review and meta-analysis. *European Spine Journal*.

[15] Khan N. R., Clark A. J., Lee S. L., Venable G. T., Rossi N. B., Foley K. T. (2015). Surgical outcomes for minimally invasive vs open transforaminal lumbar interbody fusion: an updated systematic review and meta-analysis. *Neurosurgery*.

[16] Matz P. G., Meagher R., Lamer T. (2016). Guideline summary review: an evidence-based clinical guideline for the diagnosis and treatment of degenerative lumbar spondylolisthesis. *The Spine Journal*.

[17] Herkowitz H. N., Kurz L. T. (1991). Degenerative lumbar spondylolisthesis with spinal stenosis: a prospective study comparing decompression with decompression and intertransverse process arthrodesis. *Journal of Bone and Joint Surgery*.

[18] McAfee P. C., DeVine J. G., Chaput C. D. (2005). The indications for interbody fusion cages in the treatment of spondylolisthesis: analysis of 120 cases. *Spine*.

[19] Liu X., Wang Y., Qiu G., Weng X., Yu B. (2014). A systematic review with meta-analysis of posterior interbody fusion versus posterolateral fusion in lumbar spondylolisthesis. *European Spine Journal*.

[20] Min J.-H., Jang J.-S., Lee S.-H. (2007). Comparison of anterior- and posterior-approach instrumented lumbar interbody fusion for spondylolisthesis. *Journal of Neurosurgery: Spine*.

[21] Moher D., Liberati A., Tetzlaff J., Altman D. G. (2009). Preferred reporting items for systematic reviews and meta-analyses: the PRISMA statement. *Physical Therapy*.

[22] Slim K., Nini E., Forestier D., Kwiatkowski F., Panis Y., Chipponi J. (2003). Methodological index for non-randomized studies (*MINORS*): development and validation of a new instrument. *ANZ Journal of Surgery*.

[23] Zeng X., Zhang Y., Kwong J. S. W. (2015). The methodological quality assessment tools for preclinical and clinical studies, systematic review and meta-analysis, and clinical practice guideline: a systematic review. *Journal of Evidence-Based Medicine*.

[24] Ghahreman A., Ferch R. D., Rao P. J., Bogduk N. (2010). Minimal access versus open posterior lumbar interbody fusion in the treatment of spondylolisthesis. *Neurosurgery*.

[25] Wang J., Zhou Y., Zhang Z. F., Li C. Q., Zheng W. J., Liu J. (2010). Comparison of one-level minimally invasive and open transforaminal lumbar interbody fusion in degenerative and isthmic spondylolisthesis grades 1 and 2. *European Spine Journal*.

[26] Archavlis E., Carvi y Nievas M. (2013). Comparison of minimally invasive fusion and instrumentation versus open surgery for severe stenotic spondylolisthesis with high-grade facet joint osteoarthritis. *European Spine Journal*.

[27] Kotani Y., Abumi K., Ito M., Sudo H., Abe Y., Minami A. (2012). Mid-term clinical results of minimally invasive decompression and posterolateral fusion with percutaneous pedicle screws versus conventional approach for degenerative spondylolisthesis with spinal stenosis. *European Spine Journal*.

[28] Parker S. L., Mendenhall S. K., Shau D. N. (2014). Minimally invasive versus open transforaminal lumbar interbody fusion for degenerative spondylolisthesis: comparative effectiveness and cost-utility analysis. *World Neurosurgery*.

[29] Hibbs R. A. (1911). An operation for progressive spinal deformities. *New York Medical Journal*.

[30] Kim K.-T., Lee S.-H., Lee Y.-H., Bae S.-C., Suk K.-S. (2006). Clinical outcomes of 3 fusion methods through the posterior approach in the lumbar spine. *Spine*.

[31] Phillips F. M., Slosar P. J., Youssef J. A., Andersson G., Papatheofanis F. (2013). Lumbar spine fusion for chronic low back pain due to degenerative disc disease: a systematic review. *Spine*.

[32] Zhang Q., Yuan Z., Zhou M., Liu H., Xu Y., Ren Y. (2014). A comparison of posterior lumbar interbody fusion and transforaminal lumbar interbody fusion: a literature review and meta-analysis. *BMC Musculoskeletal Disorders*.

[33] Kim K.-T., Lee S.-H., Suk K.-S., Bae S.-C. (2006). The quantitative analysis of tissue injury markers after mini-open lumbar fusion. *Spine*.

[34] Kim D.-Y., Lee S.-H., Chung S. K., Lee H.-Y. (2005). Comparison of multifidus muscle atrophy and trunk extension muscle strength: percutaneous versus open pedicle screw fixation. *Spine*.

[35] Foley K. T., Gupta S. K., Justis J. R., Sherman M. C. (2001). Percutaneous pedicle screw fixation of the lumbar spine. *Neurosurgical Focus*.

[36] Lee K. H., Yeo W., Soeharno H., Yue W. M. (2014). Learning curve of a complex surgical technique: minimally invasive transforaminal lumbar interbody fusion (MIS TLIF). *Journal of Spinal Disorders and Techniques*.

[37] Nandyala S. V., Fineberg S. J., Pelton M., Singh K. (2014). Minimally invasive transforaminal lumbar interbody fusion: one surgeon's learning curve. *Spine Journal*.

[38] Sulaiman W. A., Singh M. (2014). Minimally invasive versus open transforaminal lumbar interbody fusion for degenerative spondylolisthesis grades 1-2: patient-reported clinical outcomes and cost-utility analysis. *Ochsner Journal*.

[39] Quraishi N. A., Rampersaud Y. R. (2013). Minimal access bilateral transforaminal lumbar interbody fusion for high-grade isthmic spondylolisthesis. *European Spine Journal*.

[40] Goyal N., Wimberley D. W., Hyatt A. (2009). Radiographic and clinical outcomes after instrumented reduction and transforaminal lumbar interbody fusion of mid and high-grade isthmic spondylolisthesis. *Journal of Spinal Disorders and Techniques*.

[41] Jia P. L., Zhang P. F., Li H. D., Zhang L. H., Chen Y., Zhang M. M. (2014). Literature review on clinical decision support system reducing medical error. *Journal of Evidence-Based Medicine*.

