# Supplementary material for: The Outcomes of Minimally Invasive versus Open Posterior Approach Spinal Fusion in Treatment of Lumbar Spondylolisthesis: The Current Evidence from Prospective Comparative Studies
Source: Biomed Res Int. 2017 Jan 5;2017:8423638. doi: 10.1155/2017/8423638 (PMC5244007; doi:10.1155/2017/8423638)

**Figure S1**: The results of sensitivity analysis for estimated blood loss.


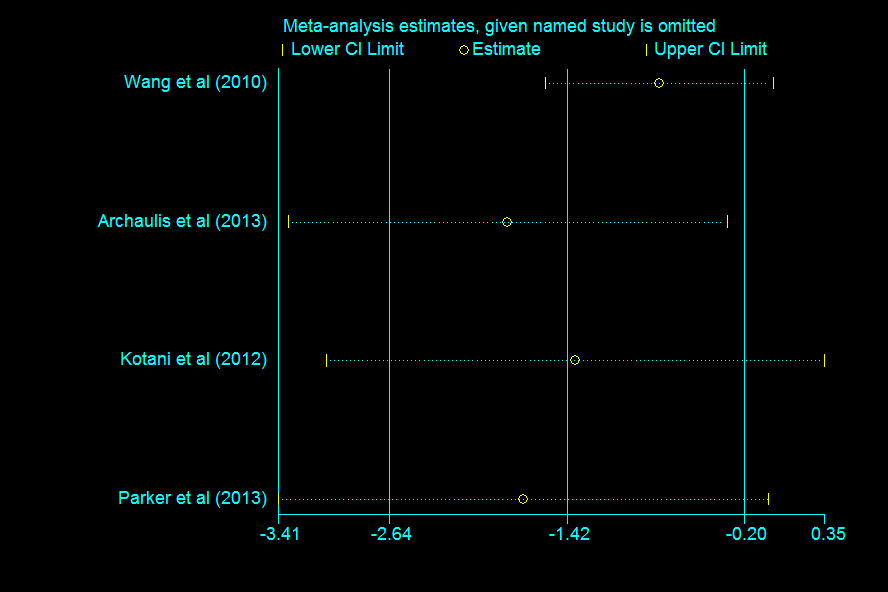


**Figure S2**: The results of sensitivity analysis for length of hospital stay.


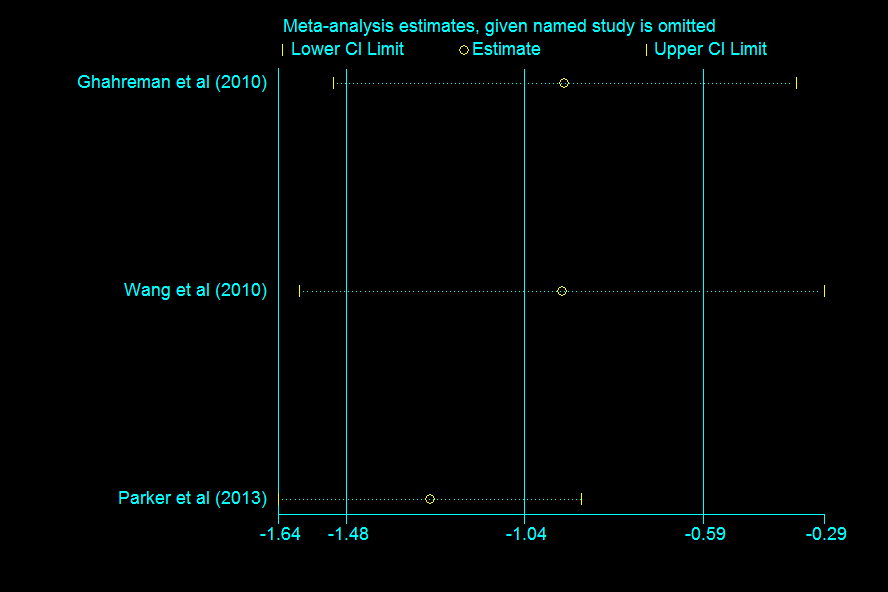


**Figure S3**: The results of sensitivity analysis for ODI.


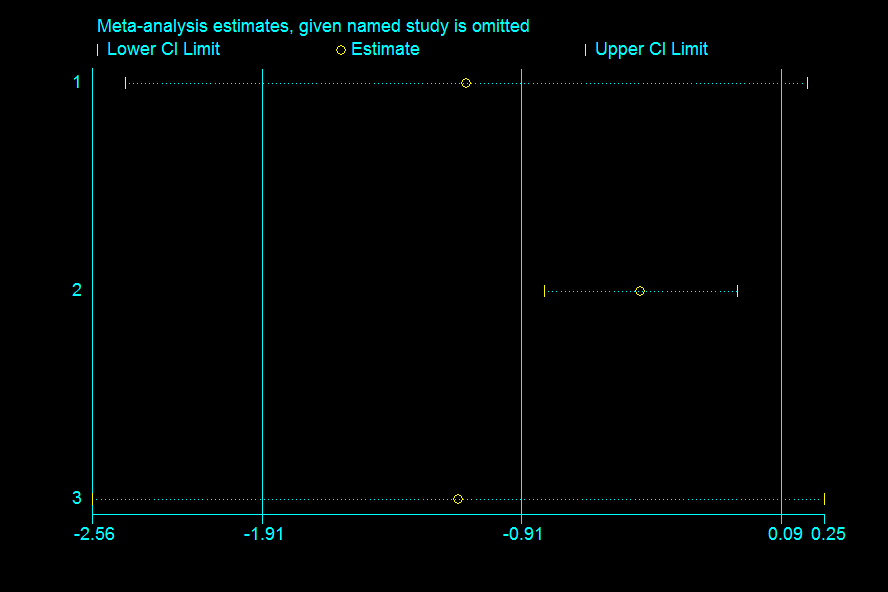

Supplement: Supplementary file 1 — Checklist S1: PRISMA 2009 checklist. Table S1: The developed search strategy performed in database of Pubmed. Its already right here. Figure S1–3: The results of sensitivity analysis. [file 8423638.f1.zip › Supplementary Figure S1-3 the results of sensitivity analysis.docx]
