# Supplementary material for: The Outcomes of Minimally Invasive versus Open Posterior Approach Spinal Fusion in Treatment of Lumbar Spondylolisthesis: The Current Evidence from Prospective Comparative Studies
Source: Biomed Res Int. 2017 Jan 5;2017:8423638. doi: 10.1155/2017/8423638 (PMC5244007; doi:10.1155/2017/8423638)
Supplement: Supplementary file 1 — Checklist S1: PRISMA 2009 checklist. Table S1: The developed search strategy performed in database of Pubmed. Its already right here. Figure S1–3: The results of sensitivity analysis. [file 8423638.f1.zip › Table S1.docx]

| **Table S1**: The developed search strategy performed in database of Pubmed. | |
| --- | --- |
| No | Search terms |
| #1 | posterior lumbar interbody fusion |
| #2 | transforaminal lumbar interbody fusion |
| #3 | posterolateral lumbar fusion |
| #4 | posterior lumbar fusion |
| #5 | posterior lumbar arthrodesis |
| #6 | minimally invasive lumbar fusion |
| #7 | minimally invasive fusion |
| #8 | #1 or #2 or #3 or #4 or #5 or #6 or #7 |
| #9 | spondylolisthesis |
| #10 | isthmic spondylolisthesis |
| #11 | degenerative spondylolisthesis |
| #12 | #9 or #10 or #11 |
| #13 | #8 and #12 |
